# Supplementary material for: Efficacy of a chemiluminescence-based urinary LAM (AIMLAM) assay in the diagnosis of active TB in Chinese children
Source: Microbiol Spectr. 2026 Mar 30;14(5):e03571-25. doi: 10.1128/spectrum.03571-25 (PMC13141949; doi:10.1128/spectrum.03571-25)
Supplement: Supplemental figure and table — Figure S1 and Tables S1 to S5. [file spectrum.03571-25-s0001.pdf]

Table S1. AIMLAM performances in active TB with bootstrapping with optimism correction when the cutoff was set at 0.1, 0.13, and 0.14, and the performance when the cutoff was 0.4.

	AIMLAM									
**Performance Measurement**	**cutoff=0.4**	**cutoff=0.14**		**Optimism (%)**	**cutoff=0.13**		**Optimism (%)**	**cutoff=0.10**		**Optimism (%)**
		**uncorrected**	**corrected**		**uncorrected**	**corrected**		**uncorrected**	**corrected**	
**Sensitivity% (95%CI),n/N**	52.2(46.6-57.6),173/331	67.4(62.1-72.1),223/331	67.4(62.2-72.4)	0.1	68.6(63.5-73.4),227/331	68.5(63.3-73.7)	-0.1	70.7 (65.4-76.0),234/331	70.8(65.4-76.0)	-0.1
**Specificity% (95%CI),n/N**	91.9(87.6-94.9),228/248	80.6(75.8-85.4),200/248	80.6(75.5-85.2)	0.1	79.4(74.3-84.6),197/248	79.5(74.4-84.7)	-0.1	77.4 (71.9-82.5),192/248	77.5(71.9-82.5)	-0.1
**PPV% (95%CI),n/N**	89.6(83.6-93.0),173/193	82.3(77.4-86.8),223/271	82.2(77.4-86.8)	0.1	81.7(77.1-86.4),227/278	81.8(77.1-86.4)	-0.1	80.7 (75.8-85.1),234/290	80.8(75.8-85.1)	-0.1
**NPV% (95%CI),n/N**	59.1(54.0-64.0),228/386	64.9(59.7-70.2),200/308	64.9(59.7-70.2)	0.1	65.4(60.2-70.8),197/301	65.5(60.2-70.8)	-0.1	66.4 (60.7-71.7),192/288	66.4(60.7-71.7)	-0.0

Table S2. Xpert and Ultra performances in Pulmonary TB and Extrapulmonary TB.

Group	Sensitivity% (95%CI),n/N		PPV% (95%CI),n/N		NPV% (95%CI),n/N		Specificity% (95%CI),n/N	
	Xpert	Ultra	Xpert	Ultra	Xpert	Ultra	Xpert	Ultra
**Pulmonary TB**	13.1(7.5-21.8),13/99	27.8(18.6-39.2),22/79	100.0(71.7-100.0),13/13	95.7(76.0-99.8),22/23	59.0(52.1-65.7),124/210	95.7(76.0-99.8),22/23	100.0(96.3-100.0,124/124	95.7(76.0-99.8),22/23
**Extrapulmonary TB**	38.3(24.9-53.6),18/47	47.0(34.7-69.6),31/66	100.0(78.1-100.0),18/18	96.9(82.0-99.8),31/32	81.0(73.7-86.7),124/153	96.9(82.0-99.8),31/32	100.0(96.3-100.0),124/124	96.9(82.0-99.8),31/32

Table S3. AIMLAM and Xpert performances in smear-positive and smear-negative samples.

Performance Measurement	AIMLAM		Xpert	
	**Smear-Positive (N=16)**	**Smear-Negative (N=308)**	**Smear-Positive (N=11)**	**Smear-Negative (N=138)**
**Sensitivity% (95%CI),n/N**	68.8 (41.5-87.9),11/16	52.6 (46.9-58.3),162/308	45.5 (18.1-75.4),5/11	22.4 (16.0-30.5),31/138
**Specificity% (95%CI),n/N**	91.9 (87.6-94.9),228/248	91.9 (87.6-94.9),228/248	91.9 (87.6-94.9),228/248	91.9 (87.6-94.9),228/248
**PPV% (95%CI),n/N**	35.5 (19.8-54.6),11/31	89.0 (83.3-93.0),162/182	20.0 (7.6-41.3),5/25	60.8 (46.1-73.8),31/51
**NPV% (95%CI),n/N**	97.8 (94.8-99.2),228/233	61.0 (55.8-65.9),228/374	97.4 (94.2-99.0),228/234	68.1 (62.7-73.0),228/335

Table S4. Diagnostic accuracy of combined assays for pediatric tuberculosis.

Performance Measurement	Combined AIMLAM and Xpert	Combined AIMLAM and Ultra	*P* value	Kappa value
**Active TB**				
Sensitivity% (95%CI),n/N	56.2 (48.1-64.0),82/146	64.1 (56.1-71.5),93/145	0.188	0.591
Specificity% (95%CI),n/N	95.2 (89.8-97.7),118/124	99.1 (94.9-99.8),106/107	0.126	
PPV% (95%CI),n/N	93.2 (85.9-96.8),82/88	98.9 (94.2-99.8),93/94	0.059	
NPV% (95%CI),n/N	64.8 (57.7-71.4),118/182	67.1 (59.4-73.9),106/158	0.731	
**Bacteriologically confirmed TB**				
Sensitivity% (95%CI),n/N	69.2 (50.0-83.5),18/26	82.4 (59.0-93.8),14/17	0.480	0.928
Specificity% (95%CI),n/N	95.2 (89.8-97.8),118/124	99.1 (95.0-99.8),106/107	0.126	
PPV% (95%CI),n/N	75.0 (55.1-88.0),18/24	93.3 (70.2-98.8),14/15	0.215	
NPV% (95%CI),n/N	93.7 (88.0-96.7),118/126	97.2 (92.2-99.1),106/109	0.229	
**Clinical diagnosed TB**				
Sensitivity% (95%CI),n/N	53.3 (44.4-62.0),64/120	62.2 (53.5-70.2),79/127	0.197	0.575
Specificity% (95%CI),n/N	95.2(89.8-97.8),118/124	99.1(94.9-99.8),106/107	0.126	
PPV% (95%CI),n/N	91.4 (82.5-96.0),64/70	98.8 (93.3-99.8),14/15	0.050	
NPV% (95%CI),n/N	67.8 (60.6-74.3),118/174	68.8 (61.1-75.6),106/154	0.905	

Table S5. Complete 2×2 table with three different thresholds（cutoff=0.1,0.13 and 0.14）

	Active TB											
	**Non-TB**	**ATB**	**Total**	**Non-TB**	**ATB**	**Total**	**Non-TB**	**ATB**	**Total**	**Non-TB**	**ATB**	**Total**
	**cutoff=0.4**			**cutoff=0.14**			**cutoff=0.13**			**cutoff=0.1**		
**Test Positive**	20	173	193	48	223	270	51	227	282	56	234	291
**Test Negative**	228	158	386	200	108	309	197	104	297	192	97	288
**Total**	248	331		248	331		248	331		248	331	

Sample Testing Procedure

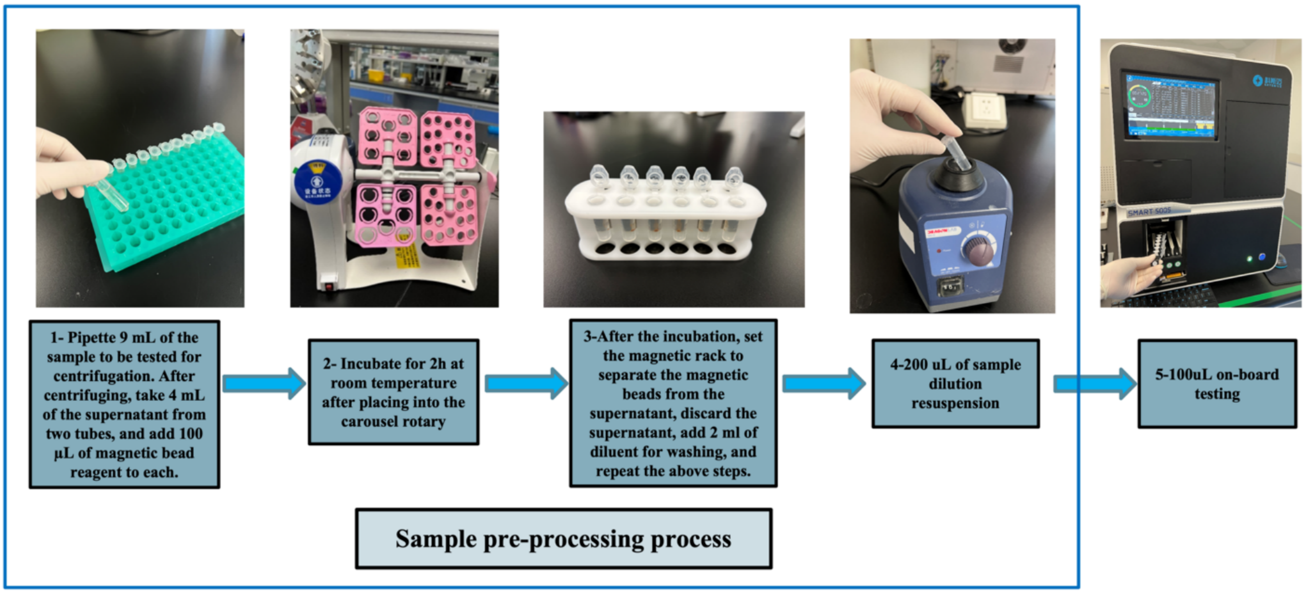

Figure S1. AIMLAM test procedure.
